# Perfusion index: Could this be a new triage tool for upper gastrointestinal system bleeding in the emergency department? A prospective cohort study

**DOI:** 10.1590/1516-3180.2021.0106.R1.0904221

**Published:** 2021-10-11

**Authors:** Basak Toptas Firat, Muge Gulen, Salim Satar, Ahmet Firat, Selen Acehan, Cem Isikber, Adem Kaya, Gonca Koksaldi Sahin, Haldun Akoglu

**Affiliations:** I MD. Emergency Physician, Department of Emergency Medicine, Adana City Training and Research Hospital, Adana, Turkey.; II MD, PhD. Associate Professor, Department of Emergency Medicine, Adana City Training and Research Hospital, Adana, Turkey.; III MD, PhD. Associate Professor, Department of Emergency Medicine, Adana City Training and Research Hospital, Adana, Turkey.; IV MD. Internal Medicine Physician, Department of Intensive Care Unit, Cukurova University School of Medicine, Adana, Turkey.; V MD. Emergency Physician, Department of Emergency Medicine, Adana City Training and Research Hospital, Adana, Turkey.; VI MD. Emergency Physician, Department of Emergency Medicine, Adana City Training and Research Hospital, Adana, Turkey.; VII MD. Emergency Physician, Department of Emergency Medicine, Adana City Training and Research Hospital, Adana, Turkey.; VIII MD. Emergency Physician, Department of Emergency Medicine, Adana City Training and Research Hospital, Adana, Turkey.; IX MD, PhD. Professor, Department of Emergency Medicine, Marmara University School of Medicine, Istanbul, Turkey.

**Keywords:** Emergencies, Perfusion index, Upper gastrointestinal tract, Rockall score, Upper gastrointestinal system bleeding, Transfusion, Emergency department, Mortality

## Abstract

**BACKGROUND:**

Many scoring systems for predicting mortality, rebleeding and transfusion needs among patients with upper gastrointestinal bleeding (UGIB) have been developed. However, no scoring system can predict all these outcomes.

**OBJECTIVE:**

To show whether the perfusion index (PI), compared with the Rockall score (RS), helps predict transfusion needs and prognoses among patients presenting with UGIB in emergency departments. In this way, critical patients with transfusion needs can be identified at an early stage.

**DESIGN AND SETTING:**

Prospective cohort study in an emergency department in Turkey, conducted between June 2018 and June 2019.

**METHODS:**

Patients’ demographic parameters, PI, RS, transfusion needs and prognosis were recorded.

**RESULTS:**

A total of 219 patients were included. Blood transfusion was performed in 174 patients (79.4%). The PI cutoff value for prediction of the need for blood transfusion was 1.17, and the RS cutoff value was 5. The area under the curve (AUC) value for PI (AUC: 0.772; 95% confidence interval, CI: 0.705-0.838; P < 0.001) was higher than for RS (AUC: 0.648; 95% CI: 0.554-0.741; P = 0.002). 185 patients (84.5%) were discharged, and 34 patients (15.5%) died. The PI cutoff value for predicting mortality was 1.1, and the RS cutoff value was 7. The AUC value for PI (AUC: 0.743; 95% CI: 0.649-0.837; P < 0.001) was higher than for RS (AUC: 0.725; 95% CI: 0.639-0.811; P < 0.001).

**CONCLUSION:**

PI values for patients admitted to emergency departments with UGIB on admission can help predict their need for transfusion and mortality risk.

## INTRODUCTION

Upper gastrointestinal bleeding (UGIB) is a common and life-threatening reason for admission to emergency departments. Despite all improvements to diagnoses and treatments, the mortality rate is still 4%-14%, and the rebleeding rate is 10%-30%.^1^^,^^2^^,^^3^ Many scoring systems that have been developed have been reported to be useful in predicting mortality, rebleeding and transfusion needs among patients with UGIB. Performing triage among patients with correct risk scores helps both to increase care efficiency and to identify patients with poor prognoses.^4^

One of the most widely used of these scores is the Rockall score (RS) system. The RS system is based on endoscopic and pre-endoscopic findings such as age, comorbidity, shock status, endoscopic diagnosis, and major new signs of bleeding.^5^ The aim in using the RS is to be able to discharge low-risk patients by performing early endoscopy, thereby shortening their length of stay in the hospital and reducing treatment costs.^6^ Furthermore, the RS has also been shown to predict the risks of rebleeding and mortality among hospitalized patients.^7^

The perfusion index (PI) is obtained by indirectly measuring pulsatile arterial flow in a specific area such as a hand or a finger, through a noninvasive method for ascertaining peripheral perfusion status. It is expressed as a percentage of the pulsatile current signal relative to the non-pulsatile current signal.^8^ It is used in many scenarios within medical practice today.

Evaluating whether anesthesia is successful or not during general, epidural or local anesthesia provides further information for identifying critical patients in neonatal intensive care units.^9^^,^^10^ Studies have shown that PI is more sensitive than oxygen saturation or pulse rate for predicting disease severity.^10^^,^^11^ In addition to studies conducted in intensive care units, PI has previously been studied in emergency departments to determine the severity of dehydration in patients with acute gastroenteritis and to predict occurrences of hypovolemic shock in trauma patients.^14^^,^^15^ However, the clinical significance of PI in patients with UGIB has not been adequately studied.

## OBJECTIVE

In this study, we aimed to compare the predictive strength of RS and PI with regard to the need for transfusion and prognosis among patients presenting with UGIB who were admitted to an emergency department.

## METHODS

This was a cohort study and was conducted prospectively. Patients with UGIB who were admitted to the emergency department of a tertiary-level hospital in Turkey between June 1, 2018, and June 1, 2019, were included in the study. The study was started after receiving approval from the Cukurova University Medical Faculty Non-Interventional Clinical Research Ethics Committee, through its meeting number 77 and decision 6, dated May 4, 2018.

### Patients

A total of 219 patients were included in this study. The following inclusion criteria were used: (1) cases of upper gastrointestinal bleeding, presented with complaints of hematemesis, melena, hematochezia, dizziness, syncope or blood from nasogastric tube aspiration; (2) cases of gastrointestinal bleeding confirmed by means of upper gastrointestinal endoscopy; (3) age ≥ 18 years. The following situations were taken to be exclusion criteria: (1) endoscopy could not be performed (unavailability, refusal or intolerance); (2) UGIB was not detected on endoscopy; (3) lower gastrointestinal tract bleeding was detected on colonoscopy; (4) coinfection with UGIB or (5) incomplete medical records.

### Sample size

The sample size was estimated by means of the G*Power for Mac OS X software (version 3.1.9.2; Universität Dusseldorf, Germany). Accordingly, with a type-1 error of 5%, type-2 error of 5% (power 95%) and two-sided analysis, the sample size was determined as 168 patients. Considering the possibility of protocol bias, addition of 10% to the number of patients in each arm was planned. Hence, 185 was determined as the minimum number of volunteers to be included.

### Data collection and measurements

The patients’ age, gender, comorbidities, laboratory parameters, PI, RS, endoscopy findings, amount of transfusion performed, length of hospital stay, development of rebleeding, surgical requirements and prognosis were recorded in the data collection form.

Blood transfusion decisions were planned according to the patients’ hemodynamic status and blood hemoglobin levels. The hemoglobin threshold for transfusion was 7 g/dl, with a target range for the post-transfusion hemoglobin level of 7 to 9 g/dl.^16^ The target hemoglobin level was > 9 g/dl in patients with cardiovascular disease and 8 g/dl in patients with portal hypertension. Red blood cell transfusion was considered if hemodynamic instability was observed despite appropriate fluid resuscitation, even if the hemoglobin level was normal.^16^^,^^17^

The RS, including age, shock status, comorbidities and endoscopic parameters, was calculated for each patient at the time when first admitted to the emergency department. This scoring system divides patients into low, medium and high-risk categories regarding rebleeding and mortality: 0-2 points constitute the low-risk category; 3-4 points, medium-risk; and ≥ 5 points, high-risk (minimum 0, maximum 11).^5^^,^^6^

The PI was measured in the supine position, noninvasively from the patient’s index fingers with the aid of a probe coupled to a Masimo Radical-7 Pulse CO-Oximeter (Masimo Corporation, Irvine, United States), at the time of admission. PI was measured for two minutes (baseline value) after signal stabilization.

### Primary outcome

The primary outcome for this study was the mortality rate observed during the hospital stay. The secondary outcomes were the need for red blood cell transfusion and the length of hospital stay.

### Statistical analysis

The Statistical Package for the Social Sciences (SPSS) software, version 22 (SPSS Inc, Chicago, Illinois, United States) was used for the statistical evaluation of the data obtained in the study. Descriptive statistics, consisting of the mean and standard deviation, were calculated for variables with normal distribution; and the minimum, maximum, median and 25%-75% percentiles were presented for variables that did not show normal distribution.

Categorical variables were compared using the chi-square test when the variables were normally distributed. Student’s t test was used for comparisons of pairs of groups. The Mann-Whitney U test was used when the variables were not normally distributed. Pearson’s correlation analysis was used to explain relationships between pairs of parametric numerical variables. The receiver operating characteristic (ROC) curve was used to evaluate the accuracy of the RS and PI in measuring hospital mortality and the need for transfusion. The Youden index, taken from the point of highest sensitivity and specificity on the ROC curve, was used to determine the cutoff value.

In investigating diagnostic test accuracy, the sensitivity and specificity parameters were calculated with 95% confidence intervals (CI) and were presented in a table. Binomial logistic regression analysis was used to reveal independent variables for predicting the prognosis. P-values < 0.05 were taken to be statistically significant.

## RESULTS

### Population characteristics

During the study period, 361 patients presented with gastrointestinal bleeding. Among these, 142 patients were excluded for the following reasons: patients with lower GIS bleeding (n = 84); endoscopy was refused or intolerance was shown (n = 26); presentation of infection (n = 32). Thus, in the end, 219 patients were included in the study based on the inclusion criteria. 69.9% (n = 153) of these patients were male and 30.1% (n = 66) were female. The mean age of the patients was 64.14 ± 17.2 years (minimum 22 to maximum 95 years).

The most common symptom was hematemesis, in 43.8% of the patients included. The most common comorbidity was coronary artery disease, in 34.7%. Among the medications thought to cause bleeding, the most commonly used drug was antiaggregant, used by 37.4%. The most common endoscopic diagnosis was a duodenal ulcer (43.4%). Endoscopic hemostasis therapy was applied to 73.3% of all the patients. The most common endoscopic procedure performed was sclerotherapy (63%). Only red blood cell transfusion was performed, in 51.5% of the patients (n = 113), and both erythrocyte and fresh frozen plasma transfusion were performed in 27.8% (n = 61) of the patients.

While 84.5% (n = 185) of the patients were discharged, 15.5% (n = 34) died. It was found that 15.1% (n = 33) of the patients had rebleeding during their follow-up. While 63.6% (n = 21) of the patients who experienced rebleeding died, 36.4% (n = 12) were discharged (P < 0.001). Surgery was required in 4.1% of all the patients (n = 9) at the time of admission. While four patients who need surgery died, five patients were discharged (P = 0.014). The characteristic features of the patients according to their transfusion needs and prognosis are presented in Table 1.

**Table 1.** Characteristics of patients according to the requirement for transfusion and hospital mortality

	Transfused patients n = 174	Non-transfused patients n = 45	P-values	Survivors n = 185	Non-survivors n = 34	P-values
**Age (years)** median ± SD	66.0 ± 16.2	56.8 ± 19.6	**0.005**	63.1 ± 17.5	69.6 ± 15.2	**0.046**
Sex, n						
Female	58	8	**0.046**	52	14	0.155
Male	116	37		133	20	
Symptoms, n						
Hematemesis	72	24	0.178	78	18	0.264
Melena	56	16	0.726	63	10	0.694
Syncope	12	1	0.476	13	0	0.228
Hematochezia	11	1	0.467	8	4	0.96
Dizziness	22	3	0.429	23	2	0.384
Other	13	2	0.741	14	1	0.476
Comorbidity, n						
CAD	64	12	0.224	65	11	0.846
HT	53	9	0.196	53	9	1.00
Malignancy	18	0	**0.028**	7	11	**< 0.001**
CLS	8	5	0.148	12	1	0.697
CKD	8	3	0.700	8	3	0.383
COPD	4	3	0.154	5	2	0.298
DM	32	7	0.827	36	3	0.220
No comorbidity	47	21	**0.018**	61	7	0.223
Vital signs and laboratory parameters						
MAP (mmHg)	79.5 ± 15.9	85.3 ± 15.6	**0.031**	81.8 ± 14.8	74.9 ± 20.6	0,073
Pulse (beat/min)	97.5 ± 19.7	92.3 ± 15.7	0.09	95.5 ± 17.9	101.4 ± 23.6	0,175
Hemoglobin (g/dl)	8.4 ± 2.3	12.0 ± 2.1	**< 0.001**	9.3 ± 2.6	8.4 ± 2.7	0,103
BUN (mg/dl)	52.7 ± 32.3	34.2 ± 16.3	**< 0.001**	46.7 ± 27.7	60.6 ± 41.5	0,070
Creatinine (mg/dl)	1.3 ± 1.3	1 ± 0.9	**0.001**	1.1 ± 0.9	2 ± 2.0	**0,020**
Lactate (mmol/l)	3.0 ± 2.7	2.0 ± 1.0	**< 0.001**	2.4 ± 1.6	5.1 ± 4.4	**0.001**
Medications, n						
No medications	63	23	0.087	77	9	0.126
Antiaggregant	70	12	0.12	72	10	0.339
NSAID	31	11	0.395	36	6	1.00
Anticoagulant	19	4	1.00	18	5	0.368
Steroid	2	1	0.50	2	1	0.399
Other medications^*^	111	22	0.087	108	25	0.126
Endoscopic findings, n						
Duodenal ulcer	79	16	0.625	88	7	**0.007**
Gastric ulcer	53	18		59	12	
Esophageal varicose veins	15	6		17	4	
Esophageal erosion	10	3		9	4	
Gastric erosion	7	2		6	3	
Gastric cancer	7	0		4	3	
Angiodysplasia	2	0		0	1	
Esophageal cancer	1	0		2	0	
Endoscopic hemostasis, n						
Sclerotherapy	55	19	0.282	68	6	**0.042**
Sclerotherapy + heater probe	43	7		43	7	
Band ligation	11	5		13	3	
Sclerotherapy + hemoclips	6	3		7	2	
APC	4	2		4	2	
Sclerotherapy + APC	5	0		2	3	
No endoscopic hemostasis	50	9		48	11	
Perfusion index median [25%-75% percentile]	0.86 [0.40-1.50]	1.60 [1.20-2.70]	**< 0.001**	1.1 [0.60-1.70]	0.41 [0.18-0.90]	**< 0.001**
Rockall scores median [25%-75% percentile]	6 [5-7]	5 [3-7]	**0.002**	6 [4-7]	7 [6-8]	**< 0.001**
Rebleeding, n	33	0	**0.002**	12	21	**< 0.001**
Surgery need, n	9	0	0.119	5	4	**0.014**

CAD = coronary artery disease; HT = hypertension; CLS = chronic liver disease; CKD = chronic kidney disease; COPD = chronic obstructive pulmonary disease; DM = diabetes mellitus; MAP = mean arterial pressure; BUN = blood urea nitrogen; NSAID = non-steroidal anti-inflammatory drug.

^*^Antihypertensive, antidiabetic, bronchodilator, antidepressant drugs; APC: Argon plasma coagulation.

Bold text indicates a statistically significant difference with a P-value < 0.05.

### Perfusion index and Rockall scores with predictions for outcomes

It was found that the patients’ red blood cell transfusion needs showed a statistically significant but weak negative correlation with the PI value (r = -0.363; P < 0.001) and a statistically significant and weak correlation with the RS (r = 0.292; P < 0.001).

For patients whose hemoglobin value was above 7 on admission, the mean PI of those who needed transfusion (n = 126) during their follow-up was 1.3 ± 1.1, and the mean PI of those who did not need transfusion (n = 45) was 1.9 ± 1. The PI level of the patients who needed a transfusion was statistically significantly lower (p = 0.001).

**Figure 1.**
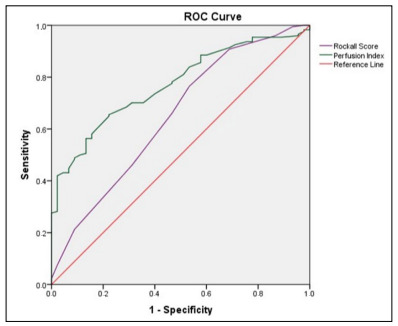
Receiver operating characteristic (ROC) curves showing comparisons of perfusion index and Rockall score for predicting need for red blood cell transfusion.

The ROC analysis graph to determine the need for red blood cell transfusion, comparing RS and PI in the patient group, is shown in Figure 1. In the analytical evaluations made, it was determined that the area under curve (AUC) value for the PI (AUC: 0.772; 95% CI 0.705 -0.838; P < 0.001) was higher than that of the RS (AUC: 0.648; 95% CI 0.554-0.741; P = 0.002). When the threshold value determined for predictability of the need for red blood cell transfusion was taken as 1.17 for PI, its sensitivity was calculated as 77.8% and specificity as 66.5%; while the sensitivity was 76.4% and specificity was 46.7% when 5 was taken as this value for RS.

The mean number of days of hospitalization for discharged patients was 5.3 ± 3.8 (minimum: 1; maximum: 23), and the mean number of days of hospitalization for patients who died was 10.4 ± 9.5 (minimum: 1; maximum: 40). There was a statistically significant difference in the number of days of hospitalization between discharged and dead patients (P < 0.001). It was determined that the patients’ hospitalization time was statistically significant but weakly correlated with RS (r = 0.293; P < 0.001), and that the higher this score was, the longer the hospitalization period also was. However, a statistically significant but very weak negative correlation was found between the PI (r = -0.160; P = 0.018) and hospitalization periods.

**Figure 2.**
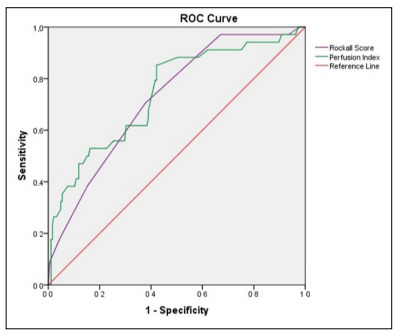
Receiver operating characteristic (ROC) curves showing comparisons of perfusion index and Rockall score for predicting hospital mortality.

The ROC analysis graph, which was produced to determine the predictive mortality properties of PI and RS, is shown in Figure 2. In the analytical evaluations conducted, it was determined that the AUC value for PI (AUC: 0.743; 95% CI: 0.649-0.837; P < 0.001) was higher than the value for RS (AUC: 0.725; 95% CI: 0.639-0.811; P < 0.001). When the threshold value determined for predicting mortality was taken as 1.1 for PI, its sensitivity was calculated as 57.8% and specificity was 85.3%; while the sensitivity was 70.6% and specificity was 62.2% when the cutoff was taken as 7 for RS.

Logistic regression analysis determined that the presence of malignancy (odds ratio, OR: 6.34; 95% CI: 1.979-20.305; P = 0.002), creatinine values > 1.4 mg/dl (OR: 1.406; 95% CI: 1.068-1.852; P = 0.015) and lactate values > 2 mmol/l) (OR: 1.328; 95% CI: 1.137-1.550; P < 0.001) were independent variables that predicted hospital mortality. However, this analysis did not show PI (OR: 0.774; 95% CI: 0.41-1.337; P = 0.323) as a statistically significant parameter in determining mortality.

## DISCUSSION

In our study, the ability of PI to predict the need for transfusion and mortality among patients with UGIB who were admitted to the emergency department was compared with the proven RS. Low PI in UGIB patients was found to be a good indicator of the ability to predict transfusion needs and mortality. We found that when PI was lower than 1.17, the need for erythrocyte suspension increased, and if it was lower than 1.1, the mortality increased significantly.

PI is obtained by dividing pulsatile arterial blood flow by the non-pulsatile flow signal, measured from an area such as the finger or toe using infrared rays.^11^ Its significant advantages are that it is noninvasive and provides continuous monitoring at the bedside that is repeatable and easy to measure.^18^ Redistribution of blood flow caused by increased vasoconstriction during hemorrhagic hypovolemia, or circulatory failure associated with low cardiac output, results in reduced skin perfusion.^19^ While vasoconstriction occurring in peripheral tissues causes a decrease in pulsatile arterial blood flow, the rate decreases because the non-pulsatile component does not decrease, which thus causes a decrease in PI.^11^ Hemoglobin and hematocrit levels are not reliable enough to predict the amount of bleeding and should not be used to rule out the presence of hypovolemic shock.^20^

Peripheral vasoconstriction caused by acute blood volume changes may be an early indicator of shock and the need for transfusion, given the rapid response to this that is seen through the PI.^15^^,^^21^ In a study evaluating the strength of the PI for predicting hypovolemic shock in trauma patients, when the cutoff value for the PI was taken as “1”, its sensitivity for predicting the need for transfusion was found to be 78% and its specificity, 97.6%.^15^ In a study in which the severity of dehydration was determined by means of the PI, among patients with acute gastroenteritis admitted to the emergency department, the PI was found to be statistically significantly lower in the moderate/severe dehydration group (PI: 1.8 (1.4-2.1)) than in the mild dehydration group (PI: 2.3 (2-2.7)).^14^ The studies conducted showed that PI could predict hypovolemia at normal blood pressure levels during the pre-shock phase.^22^

In our study, in patients with hemoglobin values above 7 on admission to the emergency department, the mean PI (1.3 ± 1.1) of the patients in need of transfusion was statistically significantly lower than the PI value of those who did not receive transfusion (1.9 ± 1). Our study also showed that PI measurement in UGIB patients could be a useful parameter for determining the need for blood transfusion. When the cutoff value of the PI was taken to be 1.17, the sensitivity was 77.8% and the specificity was 66.5%, in terms of predicting the need for transfusion. Also, according to the ROC analysis, the AUC value of PI (AUC: 0.772; P < 0.001) was found to be higher than the AUC value of RS (AUC: 0.648; P = 0.002). Therefore, we think that a low PI value may be a triage tool that will enable early detection of patients with UGIB as an effective means of triage, especially in crowded emergency departments.

Current guidelines recommend using risk stratifications to identify high-risk UGIB patients undergoing aggressive resuscitation, thereby reducing mortality and morbidity.^23^ Risk scores help predict occurrences of mortality during the hospital stay, the frequency of rebleeding, the need for transfusion and the need for hemostatic procedures through endoscopy.^1^^,^^2^^,^^3^^,^^24^ In a study comparing the RS with other risk scores, the AUC value (AUC: 0.624; P < 0.05) for the RS of patients who needed transfusion was similar to that of our study (AUC: 0.648; P = 0.002).^24^ In the same study, when the cutoff value for the RS was taken as 6, the sensitivity was 42.9% and the specificity was 90.5%, for predicting mortality.^24^

In our study, in terms of predicting mortality, the sensitivity was calculated as 70.6% and the specificity was 62.2% when the cutoff value for RS was taken as 7; while the sensitivity was 57.8% and the specificity was 85.3% when the cutoff value for PI was taken as 1.1. Also, according to the ROC analysis that was conducted to predict mortality, the AUC value for PI (AUC: 0.743; P < 0.001) was found to be higher than that of RS (AUC: 0.725; P < 0.001).

A good risk scoring system should have high sensitivity and specificity for predicting relevant outcomes, should contain easy-to-access variables, should be easy to calculate and remember and should distinguish low-risk patients from high-risk patients.^25^ Although there are many scoring systems for patients with UGIB, there is no scoring system with all of these properties. The PI has the capacity to provide continuous noninvasive monitoring regardless of laboratory parameters, in places where large numbers of patients are admitted to emergency departments and where many critical patients receive interventions at the same time. It may therefore facilitate management of critical patients with UGIB. Although there are many studies in the literature comparing risk scoring systems for use among patients with UGIB, there is also no study on the perfusion index. Our study is the first study in the literature with this feature.

### Limitations

There are some limitations to our study. First, this study was conducted at a single center in a regional referral hospital; hence, our results cannot be applied generally. Second, the decisions regarding transfusion needs were made based on clinical judgment by an individual clinician, which might have caused variability. The outcome measured in this study was whether the patient was transfused, which is different from whether the patient really needed a transfusion. Cases with hemoglobin levels between 7 and 9 could have been inappropriate for transfusion.

## CONCLUSION

The PI value of a patient admitted to the emergency department, with UGIB on admission, can help predict this patient’s need for transfusion and predict mortality. The PI has the critical advantage that it provides easy access, is noninvasive and fast and enables continuous monitoring at the bedside in the emergency department. Therefore, it can be a useful triage tool for UGIB patients admitted to crowded emergency departments. We think that prospective studies conducted with new scoring systems, in which risk scores that have proven their benefits are combined with PI, may help predict the need for transfusion and mortality.
